# Modern machine-learning can support diagnostic differentiation of central and peripheral acute vestibular disorders

**DOI:** 10.1007/s00415-020-09931-z

**Published:** 2020-06-11

**Authors:** Seyed-Ahmad Ahmadi, Gerome Vivar, Nassir Navab, Ken Möhwald, Andreas Maier, Hristo Hadzhikolev, Thomas Brandt, Eva Grill, Marianne Dieterich, Klaus Jahn, Andreas Zwergal

**Affiliations:** 1 German Center for Vertigo and Balance Disorders, Ludwig-Maximilians-University, Munich, Germany; 2 Computer Aided Medical Procedures, Technical University, Munich, Germany; 3 Department of Neurology, Ludwig-Maximilians-University, Marchioninistrasse 15, 81377 Munich, Germany; 4 Clinical Neurosciences, Ludwig-Maximilians-University, Munich, Germany; 5 Institute for Medical Information Processing, Ludwig-Maximilians-University, Biometry, and Epidemiology, Munich, Germany; 6 Munich Cluster of Systems Neurology, SyNergy, Munich, Germany; 7 Department of Neurology, Schön Klinik Bad Aibling, Munich, Germany

**Keywords:** Acute vestibular syndrome, HINTS, Machine-learning, MRI, Vestibular neuritis, Vestibular stroke

## Abstract

**Background:**

Diagnostic classification of central vs. peripheral etiologies in acute vestibular disorders remains a challenge in the emergency setting. Novel machine-learning methods may help to support diagnostic decisions. In the current study, we tested the performance of standard and machine-learning approaches in the classification of consecutive patients with acute central or peripheral vestibular disorders.

**Methods:**

40 Patients with vestibular stroke (19 with and 21 without acute vestibular syndrome (AVS), defined by the presence of spontaneous nystagmus) and 68 patients with peripheral AVS due to vestibular neuritis were recruited in the emergency department, in the context of the prospective EMVERT trial (EMergency VERTigo). All patients received a standardized neuro-otological examination including videooculography and posturography in the acute symptomatic stage and an MRI within 7 days after symptom onset. Diagnostic performance of state-of-the-art scores, such as HINTS (Head Impulse, gaze-evoked Nystagmus, Test of Skew) and ABCD^2^ (Age, Blood, Clinical features, Duration, Diabetes), for the differentiation of vestibular stroke vs. peripheral AVS was compared to various machine-learning approaches: (i) linear logistic regression (LR), (ii) non-linear random forest (RF), (iii) artificial neural network, and (iv) geometric deep learning (Single/MultiGMC). A prospective classification was simulated by ten-fold cross-validation. We analyzed whether machine-estimated feature importances correlate with clinical experience.

**Results:**

Machine-learning methods (e.g., MultiGMC) outperform univariate scores, such as HINTS or ABCD^2^, for differentiation of all vestibular strokes vs. peripheral AVS (MultiGMC area-under-the-curve (AUC): 0.96 vs. HINTS/ABCD^2^ AUC: 0.71/0.58). HINTS performed similarly to MultiGMC for vestibular stroke with AVS (AUC: 0.86), but more poorly for vestibular stroke without AVS (AUC: 0.54). Machine-learning models learn to put different weights on particular features, each of which is relevant from a clinical viewpoint. Established non-linear machine-learning methods like RF and linear methods like LR are less powerful classification models (AUC: 0.89 vs. 0.62).

**Conclusions:**

Established clinical scores (such as HINTS) provide a valuable baseline assessment for stroke detection in acute vestibular syndromes. In addition, machine-learning methods may have the potential to increase sensitivity and selectivity in the establishment of a correct diagnosis.

## Introduction

Patients with acute vertigo and dizziness account for about 4% of all visits to the emergency department (ED) [1]. Stroke is the underlying cause in 4–15% of all patients, and up to 25% of patients with the presentation of acute vestibular syndrome (AVS, defined by the presence of spontaneous nystagmus) [1, 2]. About 10% of strokes are missed at first contact [3]. Patients discharged from the ED with a suspected benign diagnosis of acute vertigo or dizziness have a 50-fold increased risk of stroke in the first week compared to matched controls [4]. Reasons for this deplorable situation are an overreliance on symptom quality and intensity as distinctive features, inadequate knowledge or application of bedside ocular motor examinations, and a blind trust in cerebral imaging results [5]. Consequently, ED physicians worldwide rank vertigo and dizziness as one of the top priorities for the development of better diagnostic algorithms [6].

Different concepts exist to differentiate peripheral and central etiologies of acute vertigo and dizziness [7, 8]. One strategy relies on a comprehensive examination of vestibular, ocular motor, and postural functions. For AVS, the HINTS test (Head Impulse, gaze-evoked Nystagmus, Test of Skew) has a high sensitivity and specificity (> 90%) for identification of stroke [9]. The diagnostic accuracy of HINTS can be further improved by video oculographic quantification of the head impulse test (vHIT) [10, 11]. Examination-based classification approaches require a profound knowledge of examination techniques and expertise in interpretation of findings. Another idea is to stratify the risk of vestibular stroke by diagnostic index tests, which aggregate information on symptom characteristics (such as symptom onset and duration, triggers, accompanying complaints) and cardiovascular risk factors (CVRF). For example, the ABCD^2^ score (Age, Blood pressure, Clinical features, Duration, Diabetes) can help to estimate the risk of vestibular stroke, but is inferior to HINTS in diagnostic accuracy [12, 13]. The advantage of index tests based on history taking is that they are easy to apply and not restricted to clinical subtypes such as AVS. Diagnostic approaches by magnetic resonance imaging (MRI) only, have a high rate of false-negative results (50% for lesions < 10 mm) in the first 48 h after symptom onset and are, therefore, not reliable during the acute stage [5, 14].

In the current study, we applied modern machine-learning algorithms to classify vestibular stroke vs. peripheral AVS due to vestibular neuritis based on a multimodal data set (including a standardized assessment of symptom features, CVRF, and detailed quantitative testing of ocular motor, vestibular, and postural functions). Machine-learning approaches were compared to state-of-the-art tests (such as HINTS, ABCD^2^) to evaluate their feasibility and value for diagnostic decision support.

## Methods

### Patient cohorts and study protocol

In total 108 patients, who were admitted to the ED of the University Hospital (LMU Munich), were included in this study and received a standardized assessment (of symptom features, CVRF, and vestibular, ocular motor and postural functions) following the EMVERT trial protocol [15]. Based on the findings of MRI (performed within 7 days after symptom onset) and videooculography (vHIT gain threshold: 0.7, refixation saccades, gaze-evoked nystagmus, skew deviation), 40 patients were diagnosed as having vestibular stroke (64.1 ± 12.2 years, 67.5% men, 19 with presentation of AVS), and 68 as having peripheral AVS due to vestibular neuritis (55.6 ± 14.6 years, 64.7% men). Classification algorithms (established index tests vs. modern machine-learning techniques) were applied post hoc to test their diagnostic accuracy for differentiation of both groups.

### Protocol approval and patient consent

The study was approved by the Ethics Committee of the University of Munich on February 23, 2015 (57–15). The study was conducted according to the Guideline for Good Clinical Practice (GCP), the Federal Data Protecting Act and the Helsinki Declaration of the World Medical Association in its current version (revision of Fortaleza, Brazil, October 2013). All subjects gave their informed, written consent to participate in the study.

### Assessment of symptom characteristics and cardiovascular risk factors

In all patients, a standardized history was taken in the ED, including the following features: symptom quality (vertigo, dizziness, double vision), symptom onset (acute, lingering), symptom duration (10–60 min, > 60 min), symptom intensity (by visual analogue scale), preceding triggers (yes, no), accompanying features (ear symptoms, central neurological symptoms), and CVRF (diabetes, high blood pressure (> 140 mmHg), nicotine abuse, atrial fibrillation, family history, prior stroke or myocardial infarction). Health-related quality of life and functional impairment was assessed by questionnaires: European Quality of Life Score—5 dimensions—5 levels (EQ-5D-5L), including subscores for anxiety, pain, activity, self-care, and mobility (ranging from 1–5 each with 5 indicating worst impairment) [16], EQ visual analogue scale (EQ-VAS) (ranging from 0–100 with 100 being the best status), Dizziness Handicap Inventory (DHI) (ranging from 0–100 points (maximum)) [17], and modified Rankin scale (mRS) (ranging from 0–6 points).

### Quantitative assessment of vestibular, ocular motor and postural functions

*Videooculography (VOG):* Vestibular and ocular motor signs were documented by VOG (EyeSeeCam®, EyeSeeTec GmbH, Munich, Germany) during the acute stage of symptoms, including nystagmus in straight ahead position (slow phase velocity (SPV) (°/sec), amplitude (°), horizontal and vertical component, with and without fixation), horizontal vestibulo-ocular reflex (VOR) function by vHIT (gain, presence of refixation saccades), gaze-evoked nystagmus (SPV (°/sec), horizontal and vertical component, lateral and vertical gaze positions), saccades (velocity (°/sec), horizontal and vertical direction), smooth pursuit (gain, horizontal and vertical direction), fixation suppression of the VOR (gain, horizontal direction), and skew deviation (cover test in six gaze positions). VOR gain was rated as pathological for values < 0.7. Suppression of spontaneous nystagmus (SPN) was positive, if the horizontal or vertical component of the SPV decreased by at least 40% on fixation.

*Testing of subjective visual vertical (SVV):* The SVV was measured by the bucket test method as described previously [18, 19]. Ten repetitions (5 clockwise/ 5 counter clockwise rotations) were performed and a mean of the deviations was calculated. The normal range was defined as 0 ± 2.5° [19].

*Posturography:* A posturographic measurement of body sway was performed using a mobile device (Wii Balance Board®, Nintendo Co. Ltd., Kyoto, Japan). Four conditions were tested: bipedal standing with eyes open/closed, upright tandem standing with eyes open/closed. For each condition, the sway pattern, normalized path length, root mean square, and peak-to-peak values in medio-lateral and anterior–posterior direction were analyzed.

### MRI protocol

The standardized protocol included whole brain and brainstem fine slice (3 mm) diffusion-weighted images (DWI), whole brain fluid attenuated inversion recovery (FLAIR)- and T2-weighted images including brainstem fine slicing (3 mm), T2*-weighted images, 3D-T1-weighted sequences (FSPGR 1 mm isovoxel) and time-of-flight angiography. All images were evaluated for the presence of ischemic stroke or bleeding by two specialized neuro-radiologists.

### Classification methods

We prospectively evaluated two established diagnostic index tests, the HINTS and ABCD^2^ clinical scores for stroke detection, to establish a baseline classification performance. We compared these baselines against the performance of various modern machine-learning techniques. The latter learn the mapping of 305 input features (from history taking, questionnaires, and instrumentation-based examinations) to the output class of stroke vs. peripheral AVS. The classification performance is quantified with three diagnostic test measures [20], namely the area-under-the-curve of a receiver-operating-characteristic (ROC-AUC), accuracy, and F1-score, defined as:

$${\text{Accuracy}}=\frac{TP+TN}{N}$$

$$\text{F1} - \text{score}= \frac{2\bullet {\text{precision}}\bullet {\text{recall}}}{{\text{precision}}+{\text{recall}}};{\text{precision}}=\frac{TP}{TP+FP};{\text{recall}}=\frac{TP}{TP+FN}$$

Here, TP/TN/FP/FN indicate the number of true-positive/true-negative/false-positive/false-negative detections, respectively, and *N* indicates the number of test samples overall. The established diagnostic index tests and each of the machine-learning techniques are described briefly in the following.

*HINTS:* The HINTS clinical scoring system aggregates a risk score for detection of vestibular stroke, as proposed in [9]. HINTS constitutes a 3-step examination, based on Head Impulse, gaze-evoked Nystagmus, and Test of Skew. HINTS indicates a central pattern, if horizontal head impulse test is normal, and/or a direction-changing nystagmus in eccentric gaze, and/or a skew deviation is detected. Consequently, in our data set we give 1 point per central HINTS item and define a HINTS score cutoff value of ≥ 1 as indicative for vestibular stroke. From this binary value for stroke diagnosis, we compute the detection accuracy and F1-score. Additionally, we perform a receiver-operator-characteristic (ROC) analysis, varying the HINTS cutoff over our data set, to obtain an area-under-the-curve (AUC) score.

*ABCD*^*2*^*:* ABCD^2^ is an aggregative scoring system for clinical detection of stroke as proposed in [21] and validated in [22]. ABCD^2^ is based on the following features: age ≥ 60 years (1 point); blood pressure ≥ 140/90 mm Hg (1 point); clinical features: unilateral weakness (2 points), speech impairment without weakness (1 point); duration ≥ 60 min (2 points) or 10–59 min (1 point); and diabetes (1 point). For stroke detection in our study, we consider ABCD^2^ scores at a cutoff value of ≥ 3. We apply this cutoff to our dataset prospectively, and obtain the accuracy and F1-score, as well as a ROC-AUC score.

*Logistic Regression (LR):* In descriptive statistics, LR is used to report the goodness-of-fit of a linear set of equations, mapping a set of input features (i.e., observations) to a binary descriptor variable (e.g., stroke indicator variable). In this work, we use LR in a prospective/predictive manner. We regularize LR with a combined L1 and L2 loss, which allows learning of a Lasso-like sparse model, while still maintaining the regularization properties of a ridge classifier [23, 24]. The balancing ratio between the L1 and L2 losses is optimized during learning as a hyper-parameter. After fitting the LR parameters to samples in a training set, we apply the fitted model to samples in a holdout test set, to obtain a logistical posterior probability of stroke. We binarize the soft decision output of LR at a posterior probability $p\left({\text{stroke}}|{\text{features}}\right)>0.5$, from which accuracy and F1-score are calculated. The AUC value is obtained by computing an ROC analysis on the probabilistic predictions for all samples.

*Random Forest (RF):* RF bundles an ensemble of decision tree (DT) models to compensate for tree overfitting [25] by vote aggregation [26]. In this work, we tune the number of DTs within the range of 5 to 50 trees towards optimal prediction performance. Due to the vote aggregation from the ensemble, an RF yields a probabilistic posterior. Accuracy, F1-score, and ROC-AUC are calculated on this posterior.

*Artificial neural network (ANN):* Computer-aided diagnosis has advanced due to the application of machine-learning techniques [27]. In particular, our own previous work [28–30], as well as numerous works in related literature [31] have demonstrated the effectiveness and modeling flexibility of ANNs for computer-aided diagnosis in medicine. Here, we apply a multilayer perceptron (MLP) with 305 input neurons, two hidden layers (128 and 64 neurons each), and two softmax-activated output neurons for classification. Due to the non-linear activation at the output layer, our ANN also yields a probabilistic posterior, allowing the calculation of accuracy, F1-score and ROC-AUC.

*Geometric matrix completion (GMC):* Geometric deep learning [32] is a novel field of deep learning, and has been introduced for computer-aided diagnosis in medicine only recently [33]. In previous work, we have shown that it is advantageous to construct multiple population graphs from meta-features of patients [34, 35]. We further proposed GMC [36] (denoted in the following as SingleGMC) to alleviate the common problem of missing values in medical data sets [37]. Recently, we have combined these ideas into multi-graph matrix completion (MultiGMC) [38]. Here, we apply both the original SingleGMC approach [36] and MultiGMC to our data set. In SingleGMC, we used a single graph and constructed it using age and ABCD^2^ scores. Graph connections are calculated based on similarity measures using age (age difference ± 5 years) and ABCD^2^ scores (± 1 score). For SingleGMC, the graph connectivity is the sum of these similarity measures. In MultiGMC, instead of taking the sum, we use them as two separate graphs. We learn separate patient representations within these two graphs (a single spectral convolutional layer per graph) and aggregate them via self-attention, before computing the classification posterior [38]. The calculation of accuracy, F1-score, and ROC-AUC is performed as for LR/RF/ANN.

The models LR, RF, and ANN were based on implementations in the scikit-learn machine-learning library [39], while GMC [36] and MultiGMC [38] are custom implementations, based on PyTorch [40].

### Statistical analysis

Compared to HINTS and ABCD^2^, which are evaluated prospectively on the entire data set, the training of machine-learning models on the entire data set would result in overfitting and an overly optimistic performance estimate. Instead, we split the data into a training set and a test set, to obtain a prospective classification performance for our investigated models. All machine-learning based classification results were thus obtained following a rigorous ten-fold cross-validation scheme [41], with stratified label sampling to account for class imbalance, and a data split ratio of 90% training vs. 10% testing data. To perform hyper-parameter tuning for all methods, we monitored the tuned model performances on a withheld validation set (10% of the training set). We compared the best-performing model to the other four models, in terms of classification accuracy by pair-wise, two-tailed, non-parametric hypothesis tests (Wilcoxon signed-rank test) at a level of significance* p* < 0.05.

Furthermore, to make the results of the machine-learning classifier more explainable, we used the RF classifier to compute, which features contribute the most towards the detection of stroke. Such analysis constitutes a fundamental technique in the domain of machine-learning interpretability [42]. Feature importance was calculated according to the Mean Decrease in Impurity (MDI) measure [43], as implemented in scikit-learn [39]. We ranked the discriminative power of features by sorting the MDI coefficients, and reported the top 10 most important features utilized by the RF during classification. For these features, univariate analysis of quantitative values was performed for patients with vestibular stroke and vestibular neuritis (% for categorical variables, mean ± SD for continuous variables). The parameters were compared between groups using either the Chi-square test or Mann–Whitney *U*-test applying a significance level of *p* < 0.05.

## Results

### Prospective evaluation of HINTS and ABCD^2^ diagnostic performance

**Fig. 1:**
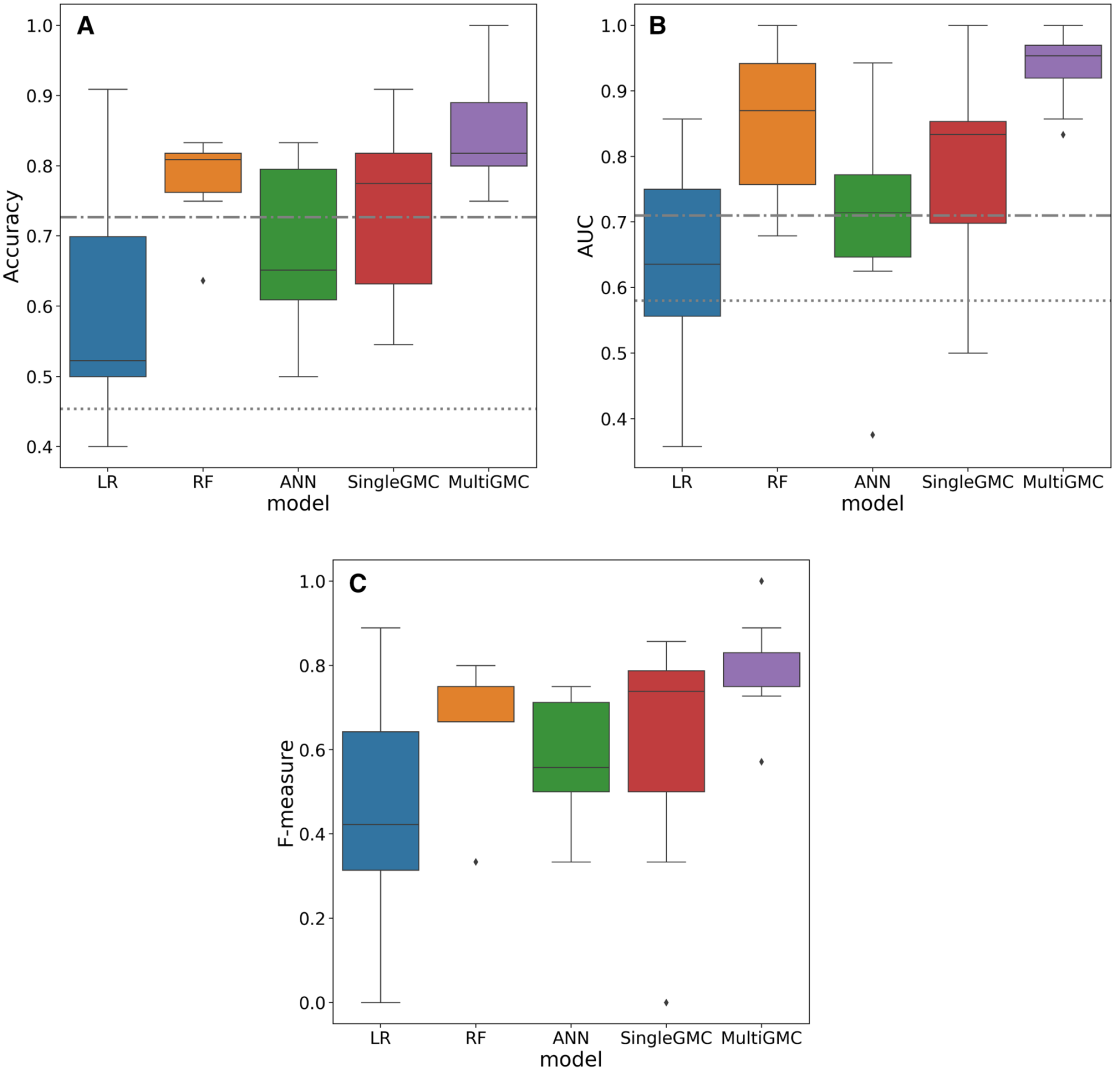
**a** Accuracy, **b** ROC-AUC, and **c** F1-score (F-measure) of five machine-learning classifiers used in this work (LR: Logistic regression, RF: Random Forest, ANN: Artificial neural network, SingleGMC: Single-graph geometric matrix completion [36], MultiGMC: Multi-graph geometric matrix completion). As a baseline comparison we additionally indicate HINTS and ABCD^2^ performances (accuracy, ROC-AUC). The prospective validation of univariate clinical scores is illustrated as grey horizontal baselines (HINTS: dash-dotted line, ABCD^2^: dotted line)

In a prospective analysis, we validated the classification scores of HINTS and ABCD^2^ for detection of all vestibular strokes (AVS and non-AVS presentation) against peripheral AVS. In our data set, HINTS was able to detect all strokes with an accuracy of 72.7%, at a ROC-AUC of 0.71. In comparison, ABCD^2^ detected stroke with a lower accuracy of 45.4%, at a ROC-AUC of 0.58. We indicate these univariate baseline methods as dashed horizontal lines in Fig. 1, to which we compare our machine-learning based models. HINTS had a diagnostic accuracy of 82.8%, at a ROC-AUC of 0.86 for stroke with AVS, and a diagnostic accuracy of 66.7%, at a ROC-AUC of 0.54 for stroke without AVS. ABCD^2^ performed with an accuracy of 37.7 (ROC-AUC of 0.59) for stroke with AVS, and 38.6% (ROC-AUC of 0.62) for stroke without AVS.

### Machine-learning models for vestibular stroke detection

The median accuracy of all machine-learning methods ranged between 52% (LR) and 82% (MultiGMC). Two models, the linear LR and the non-linear ANN, achieved lower classification accuracy than the univariate measures HINTS and ABCD^2^ for all vestibular strokes vs. peripheral AVS, while RF/SingleGMC/MultiGMC were able to achieve better accuracy (Fig. 1a). Similar results were obtained for AUC and F1-score (Fig. 1b, c). Notably, two methods (RF and MultiGMC) were also able to achieve perfect classification accuracy, F1-score, and ROC-AUC for one of the five cross-validation folds, while LR and ANN, achieved a zero (0.0) F1-score for one of five folds. In general, MultiGMC yields comparably stable results over all five folds, with consistently high accuracy, F1-score, and ROC-AUC values. Comparing machine-learning classifiers statistically, MultiGMC classifies significantly better than LR (*p* < 0.01), ANN (*p* < 0.05), and SingleGMC (*p* < 0.05), but not significantly better than RF (*p* = 0.69).

### Feature importance ranking

We used RF to rank features according to their discriminative performance. The top 10 selected features can be seen in Table 1. Features from device-based measurements such as VOG, SVV testing, and posturography, were considered as single parameters (e.g., vHIT-gain right, vHIT-gain left) or in an aggregated manner (vHIT pathological or normal based on a gain cutoff of 0.7 or presence of refixation saccades). No posturographic or SVV features were selected by the RF classifier as being among the top 10 important features. Instead, two aggregated VOG features (vHIT pathological, presence of horizontal SPN) and eight VOG-based single features were identified (e.g., vHIT gain, gaze-evoked nystagmus left, right).

**Table 1:** Top 10 most important features, ranked by RF classifier (i.e., ranked by discriminative power for classification) (left side)

Rank in RF	Feature	Feature type	Vestibular neuritis	Vestibular stroke	*P* value
1	vHIT pathological (gain < 0.7/refixation saccades)	VOG (aggregated)	100%	12.5%*	< 0.0001
2	vHIT gain (right)	VOG (single feature)	0.6 ± 0.3**	0.9 ± 0.3	< 0.0001
3	Fixation suppression of VOR gain (horizontal)	VOG (single feature)	0.03 ± 0.03	0.09 ± 0.06	< 0.0001
4	Smooth pursuit gain (downward direction)	VOG (single feature)	0.75 ± 0.17	0.67 ± 0.2	0.01
5	SPN present without fixation (horizontal)	VOG (aggregated)	95.5%***	47.5%	< 0.0001
6	SPV of SPN (0° position, vertical component)	VOG (single feature)	2.0 ± 2.5°/s	1.0 ± 1.5°/s	0.09
7	SPV of GEN (15° right, horizontal component)	VOG (single feature)	1.2 ± 1.5°/s	0.4 ± 0.6°/s	0.004
8	SPV of SPN (0° position, horizontal component)	VOG (single feature)	4.7 ± 4.0°/s	1.0 ± 1.0°/s	< 0.0001
9	SPV of GEN (15° left, horizontal component)	VOG (single feature)	1.6 ± 2.5°/s	0.3 ± 0.4°/s	0.002
10	STD of SPN amplitude (0° position, horizontal)	VOG (single feature)	2.3 ± 1.4°	1.8 ± 0.8°	0.0005

Quantification of the respective features (as % or mean ± STD) in patients with vestibular neuritis or vestibular stroke and statistical intergroup comparison (Mann–Whitney *U *test for features 2–4 and 6–10, Chi-square test for features 1 and 5) (right side).* GEN* gaze-evoked nystagmus, *SPN* spontaneous nystagmus, *SPV* slow phase velocity, *STD* standard deviation,* VOG* videooculography, *VOR* vestibulo-ocular reflex, *vHIT* video head impulse test; *vHIT was pathological in vestibular stroke lesions affecting the vestibular nucleus or medial longitudinal fascicle; **Gain is depicted for the affected side in vestibular neuritis; ***In three patients without apparent SPN, symptoms of vestibular neuritis had already started ≥ 3 days before VOG recording

Quantitative univariate analysis of the 10 most important features revealed significant intergroup differences for all but one feature (i.e., rank 6, vertical component of SPN in 0° position, *p* = 0.09) (Fig. 1). The following features discriminated best between groups: (1) vHIT was pathologic in 100% of patients with vestibular neuritis (gain: 0.6 ± 0.3 at affected side), but only in 12.5% of patients with vestibular stroke (gain: 0.9 ± 0.3) (*p* < 0.0001). (2) SPN was found more frequently in vestibular neuritis (95.5%) than in vestibular stroke (47.5%), and was more intense (horizontal SPV in 0° position: 4.7 ± 4.0°/s vs. 1.0 ± 1.0°/s) (*p* < 0.0001). (3) Fixation suppression of the VOR was abnormal in vestibular stroke (gain: 0.09 ± 0.06), but intact in vestibular neuritis (gain: 0.03 ± 0.03). SPN was suppressed by fixation in 94% of patients with vestibular neuritis. Ranking of feature importance by RF reflected clinically important parameters with significant intergroup differences.

## Discussion

Analysis of various approaches for the detection of patients with vestibular stroke (with the clinical presentation of AVS or non-AVS) vs. patients with peripheral AVS due to vestibular neuritis revealed the following findings: HINTS achieves better classification than ABCD^2^ and two of the tested machine-learning methods (LR, ANN), but is not as accurate as the more modern tested machine-learning methods (RF, Single-/MultiGMC) for differentiation of all vestibular strokes against peripheral AVS. In the following, we discuss the methodological and clinical implications of these findings.

### Comparison of the different methodological approaches

In the current study, we compared two established clinical classification scores (HINTS, ABCD^2^) to a number of machine-learning techniques, both classical methods (LR, RF, ANN) and deep learning techniques based on population-modeling with graphs (SingleGMC, MultiGMC). In terms of median accuracy and area-under-the-curve (AUC), all machine-learning classifiers outperformed the detection rate of stroke as indicated by ABCD^2^. Compared to HINTS, however, several machine-learning classifiers performed similarly (LR, ANN, SingleGMC), while only RF and MultiGMC were able to reliably outperform HINTS. For vestibular stroke with AVS, the diagnostic accuracy of HINTS was comparable to MultiGMC. From a methodological perspective, our results provide a reliable estimate of a potential prospective classification performance for future validation studies, due to the usage of a rigorous cross-validation scheme and hyper-parameter optimization of all machine-learning models. More training data in prospective studies may improve results further, as data set size is usually a limiting factor in machine-learning studies [41]. The RF models yielded satisfactory results, while deep learning models, particularly MultiGMC, were able to improve results further. In general, the possibility to incorporate a semantic population model built from disease-relevant meta-features in form of a graph is attractive from a clinical point of view. The efficacy of this approach in everyday life clinical scenarios needs to be further validated in future studies.

### Clinical implications

There is increasing discussion about the use of computer-aided diagnostic support systems in the context of complex clinical scenarios. The differentiation of central and peripheral etiologies of acute vertigo and dizziness poses such a challenge. Established diagnostic algorithms such as HINTS perform very well for AVS, which accounts for about half of acute presentations of vertigo or dizziness [9, 10]. Stroke detection remains particularly difficult, if patients have non-AVS presentations, transient or mild symptoms [3]. Therefore, in the current study we analyzed all vestibular stroke patients (AVS, non-AVS) against peripheral AVS. In our data set, ABCD^2^ had a low diagnostic performance to indicate vestibular stroke and HINTS outperformed ABCD^2^. Nevertheless, for all vestibular stroke patients (AVS, non-AVS), the diagnostic accuracy of HINTS was lower than previously reported for AVS only [9]. Modern machine-learning techniques (such as MultiGMC) had the highest diagnostic accuracy in separating vestibular stroke from peripheral AVS. Interestingly, ranking of feature importance by machine-learning algorithms (such as RF) closely resembled existing clinical experience. The top two features are derived from head impulse testing (vHIT pathologic, vHIT gain). In accordance, HIT has been previously considered the most important component of HINTS with a 18-fold stroke probability if normal in presence of SPN [44]. Two other features (ranks 7, 9) are concerned with gaze-evoked nystagmus, which is also part of HINTS. Skew deviation was not included in the 10 top features, which may be due to its low rate of manifestation (present in only about one quarter of vestibular stroke patients) [45]. Intensity of SPN was weighted prominently (ranks 5, 6, 8, 10). An additional feature with a high importance was a disturbed fixation suppression of the VOR (rank 3). This sign is regularly found in cerebellar lesions involving the uvula, pyramis, nodulus, and flocculus, which are common in patients with vestibular stroke [46, 47]. Notably, all the top-ranked features resulted from VOG examination, while SVV testing and posturography seemed to be less important. It is well-known that SVV deviation is found both in peripheral and central vestibular lesions, because it reflects a peripheral or central tone imbalance of graviceptive input originating from the vertical semicircular canals and otoliths [48]. The underrepresentation of postural parameters in our data set is in partial contrast to previous clinical studies, which have shown a high diagnostic relevance of the extent of falling tendency in AVS [49]. This discrepancy may be explained by the fact that the overall sway pattern cannot be derived from one or two features, but rather from a complex interplay of parameters [30].

## Conclusions

This feasibility study shows the potential of modern machine-learning techniques to support diagnostic decisions in acute vestibular disorders. The current algorithm is tailored for the differentiation of vestibular neuritis vs. vestibular stroke only, and heavily depends on a quantitative and comprehensive assessment of vestibular and ocular motor functions by VOG, which limits its application under everyday life conditions in the ED. Therefore, future studies should focus on tailored VOG-protocols, include other qualitative factors (like triggers, acuity of onset, accompanying symptom features), and test the validity of machine-learning approaches in larger multicenter data sets for a wider range of differential diagnoses, such as Menière’s disease and vestibular migraine.

## Abbreviations

ABCD^2^: Age, blood pressure, clinical features, duration, diabetes
ANN: Artificial neural network
AUC: Area-under-the-curve
AVS: Acute vestibular syndrome
CVRF: Cardiovascular risk factors
DT: Decision tree
DWI: Diffusion weighted images
ED: Emergency department
EMVERT: EMergency VERTigo
FLAIR: Fluid attenuated inversion recovery
GMC: Geometric matrix completion
HINTS: Head impulse, gaze-evoked nystagmus, test of skew
LR: Logistic regression
ML: Machine-learning
MLP: Multilayer perceptron
MultiGMC: Multi-graph geometric matrix completion
RF: Random forest
ROC: Receiver operating characteristic
SingleGMC: Single-graph geometric matrix completion
SPN: Spontaneous nystagmus
SPV: Slow phase velocity
STD: Standard deviation
SVV: Subjective visual vertical
vHIT: Video-based head impulse test
VOG: Videooculography
VOR: Vestibulo-ocular reflex
